# Feasibility of intermittent back-filtrate infusion hemodiafiltration to reduce intradialytic hypotension in patients with cardiovascular instability: a pilot study

**DOI:** 10.1007/s10157-016-1270-z

**Published:** 2016-04-28

**Authors:** Yutaka Koda, Ikuo Aoike, Shin Hasegawa, Yutaka Osawa, Yoichi Nakagawa, Fumio Iwabuchi, Chikara Iwahashi, Tokuichiro Sugimoto, Toshihiko Kikutani

**Affiliations:** 1 Koda Medical and Dialysis Clinic, 3748 Yoshida, Tsubame, Niigata 959-0264 Japan; 2 Koyo Medical Clinic, Konan-ku, Niigata, 950-0121 Japan; 3 Kashiwazaki General Medical Center, Kashiwazaki, Niigata 945-8535 Japan; 4 Niigata Rinko Hospital, Higashi-ku, Niigata, 950-8725 Japan; 5 Nakagawa Medical Clinic, Utsunomiya, Tochigi 321-0157 Japan; 6 Iwabuchi Clinic, Utsunomiya, Tochigi 320-0811 Japan; 7 Gyoda General Hospital, Gyoda, Saitama 361-0056 Japan; 8 Kuki General Hospital, Kuki, Saitama 346-0021 Japan; 9 Ogawa Hospital, Hiki, Saitama 355-0317 Japan

**Keywords:** Hypotension, Intermittent back-filtrate infusion, Hemodiafiltration

## Abstract

**Background:**

Intradialytic hypotension (IDH) is one of the major problems in performing safe hemodialysis (HD). As blood volume depletion by fluid removal is a major cause of hypotension, careful regulation of blood volume change is fundamental. This study examined the effect of intermittent back-filtrate infusion hemodiafiltration (I-HDF), which modifies infusion and ultrafiltration pattern.

**Methods:**

Purified on-line quality dialysate was intermittently infused by back filtration through the dialysis membrane with a programmed dialysis machine. A bolus of 200 ml of dialysate was infused at 30 min intervals. The volume infused was offset by increasing the fluid removal over the next 30 min by an equivalent amount. Seventy-seven hypotension-prone patients with over 20-mmHg reduction of systolic blood pressure during dialysis or intervention-requirement of more than once a week were included in the crossover study of 4 weeks duration for each modality. In a total of 1632 sessions, the frequency of interventions, the blood pressure, and the pulse rate were documented.

**Results:**

During I-HDF, interventions for symptomatic hypotension were reduced significantly from 4.5 to 3.0 (per person-month, median) and intradialytic systolic blood pressure was 4 mmHg higher on average. The heart rate was lower during I-HDF than HD in the later session. Older patients and those with greater interdialytic weight gain responded to I-HDF.

**Conclusions:**

I-HDF could reduce interventions for IDH. It is accompanied with the increased intradialytic blood pressure and the less tachycardia, suggesting less sympathetic stimulation occurs. Thus, I-HDF could be beneficial for some hypotension-prone patients.

**UMIN registration number:**

000013816.

## Introduction

Intradialytic hypotension (IDH) requires immediate medical intervention and/or nursing care. In addition, IDH may reduce the efficacy of dialysis, considerably increase a patient’s discomforts such as consciousness loss, and finally lead to cardiac dysfunction with myocardial stunning [1–3]. Consequently, IDH is considered an independent risk factor for patient survival [2, 4]. The high risk of IDH, methods of how to avoid it, and how to provide countermeasures are immediate issues.

One successful countermeasure is the plasma sodium biofeedback system, which can reduce the burden of IDH [5]. Fluid management, which is the other side of sodium management, has always been a central part of blood pressure stability. IDH is thought to occur most often with high fluid removal. Rapid removal of large interdialytic volume gain is associated with mortality and is very uncomfortable for patients [6]. In 2013, Mineshima and Eguchi advocated a convective type of method with cyclic back-filtration infusion, naming it as intermittent-infusion hemodiafiltration (I-HDF) [7]. It is a volume-centered approach to cardiovascular instability and an anti-fouling approach for membrane during convective therapy. I-HDF utilizes on-line-quality ultrapure dialysate which is back filtered with an automated dialysis machine. A quick and regular infusion is expected to restore blood volume, blood pressure, and peripheral circulation. Additionally, a backwash prevents hemodiafilter membrane fouling, mainly caused by low molecular weight protein [7]. We conducted our study to investigate whether this method can reduce the frequency of interventions for patients with IDH.

## Subjects and methods

### Study design and patients

The study was designed as an open, non-controlled, prospective, crossover, multicenter trial. Eight dialysis units in Japan (Niigata, Saitama, and Tochigi prefecture) participated in the study. Approval from ethics committee of Hiro Clinic Oomori, Tokyo, was obtained on 22 April, 2014 (IRB approval number: 26020), and the study was registered to UMIN (000013816).

IDH is defined in this study as systolic blood pressure falling over 20 mmHg from baseline, or presentation of symptoms associated with hypotension requiring any medical interventions, including nursing care [8, 9]. These symptoms include unconsciousness, nausea, vomiting, dyspnea, chest discomfort, muscle cramps, which require interventions such as bolus infusion, leg raising (Trendelenburg position), lowering or stopping ultrafiltration rate, discontinuation of dialysis, switching to sole ultrafiltration, prescription or modification of vasoconstrictor and antihypertensive agents, and so on.

Patients who experienced these IDH complications at least once a week were included in the study. The observation period before entry was 2 weeks, in which inclusion or exclusion was determined. All patients who took part in gave written informed consent. Eligible patients were stage 5 CKD, treated by hemodialysis thrice weekly. Dry weight was clinically and comprehensively determined based on previous history and symptoms such as dyspnea, peripheral edema and blood pressure, to the weight at which patient would remain normotensive. In all participating facilities, chest X-ray was regularly examined to measure cardio-thoracic ratio, which might help to consider appropriateness of dry weight. Patients were excluded if: (a) they had had a cardiovascular event within the past 3 months; (b) they had active inflammatory disease or malignancy; (c) they had systolic blood pressure exceeding 150 mmHg during dialysis; (d) they were treated with a dialysis membrane with a surface area less than 0.8 m^2^ or greater than 2.2 m^2^; or (e) they were on beta-2 microglobulin adsorptive columns.

I-HDF is a convective type of therapy which is approved by Japanese health care regulations and the reimbursement agency as a modified type of hemodiafiltration. Although the convective volume chosen for this study was too small to meet the definition set forth by the EUDIAL group [10], the study did utilize the high-flux hemodiafilter with internal filtration and intentional infusion of back-filtered ultrapure dialysate in a closed system. As the subjects were hypotension-prone in this study, intermittent infusion was expected to be prophylactic against hypotension. Dialysate was infused rapidly by backward filtration at a rate of 150 mL/min at 30 min intervals using a programmable system for an automated dialysis machine, either the TR-3000 MA^®^ or the TR-3300M^®^ (Toray Medical Ltd, Tokyo, Japan). To ensure patients safety during I-HDF the dialysate purity of the on-line fluid must be guaranteed by multiple endotoxin-retentive filters, and the entire system should be validated by manufacturers to limit endotoxins to below the applicable clinical detection limit and bacteria to less than 10 to minus 6 quality. In this study, hemodiafilter used for I-HDF period was specified to polysulfone TDF-M^®^ (Toray Medical Ltd, ultrafiltration rate: 46.6 mL/0.13 kPa/h with membrane area 1.5 m^2^), and the membrane area was adjusted to limit any difference less than 0.1 m^2^ from the subject’s prior dialyzer.

**Fig. 1:**
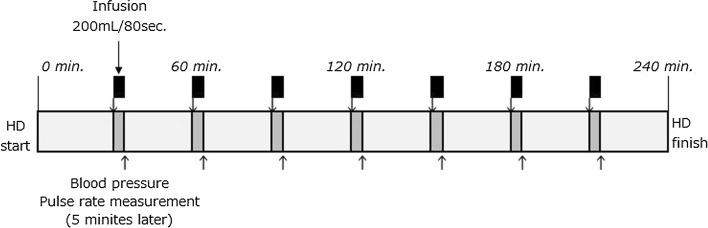
Dialysate was infused by back filtration through hemodiafilter seven times in one session at a rate of 200 mL/80 s. The procedure was performed automatically by a programmable dialysis system

As an example, in patients with 4-h sessions, each infusion bolus was 200 mL and seven infusions were given per session, providing a total of 1400 mL/session according to the protocol (see Fig. 1). Usual ultrafiltration rate based on interdialytic weight gain (IDWG) and food/water intake during session was corrected by the following equations in I-HDF (corrected ultrafiltration rate: cUFR, mL/min).

1 $${\text{Usual UFR }}\left( {{\text{mL}}/{ \hbox{min} }} \right) \, = \, \{ {\text{IDWG }}\left( {\text{mL}} \right) \, + \, \left[ {{\text{food and water intake during session }}\left( {\text{mL}} \right)} \right]\}/\left[ {{\text{session time }}\left( { \hbox{min} } \right)} \right]$$

2 $${\text{bolus infusion time }}\left( { \hbox{min} } \right) \, = \, \left[ {{\text{bolus infusion volume }}\left( {\text{mL}} \right)} \right]/\left[ {{\text{bolus infusion rate }}\left( {{\text{mL}}/{ \hbox{min} }} \right)} \right]$$

3 $${\text{corrected UFR }}\left( {{\text{mL}}/{ \hbox{min} }} \right) \, = \, \left\{ {\left[ {{\text{usual UFR}}\left( {{\text{mL}}/{ \hbox{min} }} \right)} \right] \times \left[ {{\text{interval time }}\left( { \hbox{min} } \right)} \right] + \left[ {{\text{bolus infusion volume }}\left( {\text{mL}} \right)} \right]} \right\}/\left\{ {\left[ {{\text{interval time }}\left( { \hbox{min} } \right)} \right] {-} \left[ {{\text{bolus infusion time }}\left( { \hbox{min} } \right)} \right]} \right\}$$

In this study, bolus infusion volume is 200 mL, bolus infusion rate is 150 mL/min, session time is 240 min, and interval time is 30 min.

Crossover treatment of 2 weeks of HD, 4 weeks of I-HDF, and 2 weeks of HD was conducted. Blood pressure and pulse rate was measured at 5 min after each infusion (see Fig. 1). The systolic blood pressure (SBP) of these were averaged and assumed as intradialytic SBP. The total number of interventions for IDH, the blood pressure, and the pulse rate were recorded in the last week of each study period. Data from the two HD periods (pre and post I-HDF treatment) for each patient were combined and averaged, and served as a single control.

The protocol indicated that dialysis parameters and medications related to blood pressure were to be kept the same during both treatments for the purpose of this study. However, it was allowed to change them in accordance with clinical state. If any changes were made, they were documented as interventions. The primary outcome of this study was the frequency of interventions for IDH. An intervention-decreased patient in I-HDF period is considered as an effective and improved case and defined as responder for I-HDF.

### Statistical evaluation

Results were presented as mean ± SD, median and interquartile range or IQR: 25th, 75th percentile, depending on variable distributions, and as percent for categorical variables. Analyses were conducted by Chi-squared test, Student’s *t* test (paired or non-paired), and Wilcoxon singed-rank test, where appropriate. Multivariate logistic regressions were applied to examine predictors for patients who responded to I-HDF.

All of the analyses were conducted using R version 3.1.2 software for statistical computing (R Foundation for Statistical Computing, Vienna, Austria: http://www.R-project.org/).

## Results

### Total entry and dropouts

A total of 77 patients were enrolled in the study. Table 1 summarizes the frequency and cause of dropouts. Nine patients dropped out. Six patients among them were included in the final analysis as worsened cases, though they were not necessarily exacerbated. Remaining 3 patients were not included because they withdrew before I-HDF. Thus, 68 patients completed the study and 74 patients were in final analysis.

**Table 1:** Total entry and dropouts

(a) Total entry	77									
Completion of the study	68									
Dropouts	9									

(b) Details of dropouts										
Dropout case	Dropout phase	Reason	Sex	Age (y/o)	DM	CVD	Anti-hypert. agent	Pressor agent	Session time (min)	Alb (g/dL)
1	I-HDF	Hypotension	M	74	0	1	0	0	240	3.5
2	I-HDF	Withdrawal of consent	M	78	0	0	0	0	240	3.4
3	I-HDF	Withdrawal of consent	F	60	1	1	0	0	240	3.5
4	I-HDF	Hypotension	F	88	0	1	1	0	180	3.3
5	I-HDF	Hypotension	M	70	0	1	1	0	180	3.9
6	I-HDF	Gastrointestinal hemorrhage	M	76	1	0	1	0	180	3.7
7	–	Registration only	M	86	0	1	1	0	240	3.4
8	HD	Not eligible	M	74	1	0	0	1	240	3.0
9	HD	No basic data	M	80	0	0	0	0	180	3.9
Average or *percent			M/F 7/2	76.2	33.3*	55.6*	44.4*	11.1*	213.3	3.5
SD				8.4					31.6	0.3

In the columns of DM, CVD, antihypertensive agent and pressor agent, the presence was indicated by 1, and the absence by 0. Cases 1–6 were included as worsened in the final analysis

*DM* diabetes mellitus, *CVD* cardiovascular disease, *Anti*-*hypert. agent* anti-hypertensive agent, *Alb* albumin, *y/o* years old, *SD* standard deviation

The details of dropouts are summarized in Table 1b. The characteristics of 9 dropouts, compared with 68 completed cases (dropouts vs. completed) were older age (76.2 ± 8.4 vs. 66.1 ± 11.2 years old, *P* = 0.003) and shorter session time (213.3 ± 31.6 vs. 234.3 ± 18.1 min, *P* = 0.002). Other demographics were not different.

### Baseline demographics and dialysis parameters

Baseline demographics and dialysis parameters of patients in the final analysis are summarized in Table 2.

**Table 2:** Patient demographics and dialysis parameters (final analysis including 6 dropouts)

	*n* = 74	Range
Patient demographics		
Age (years)	66.7 ± 11.3	35–87
Male (%)	52.7	
Diabetics (%)	32.4	
Vintage (years; median [IQR])	4.0[2.0–10.0]	0.3–28.0
Dry weight (kg)	54.7 ± 10.9	37.4–82.5
Interdialytic weight gain (% of DW)	4.3 ± 1.3	1.7–7.3
Serum albumin (g/dL)	3.6 ± 0.4	2.8–4.5
Cardiovascular disease (%)	44.5	
Antihypertensive agent (%)	50.0	
Pressor agent (%)	24.3	
Dialysis parameters		
Blood flow (mL/min)	200 [200–200]	160–230
Dialysate flow (mL/min; median [IQR])	500 [500–500]	500–600
Session time (min; median [IQR])	240 [240–240]	180–270
Dialysate Sodium (mEq/L; median [IQR])	140 [140–140]	140–143
Dialysate temperature (°C; median [IQR])	36 [36.0–36.5]	36.0–36.5
Dialysate buffer, bicarbonate-based; citrate/acetate	32/42	

Values are given in mean ± SD for normally distributed variables, in median [*IQR* interquartile range] for non-normally distributed and % for categorical variables

*DW* dry weight

### Primary outcome

**Fig. 2:**
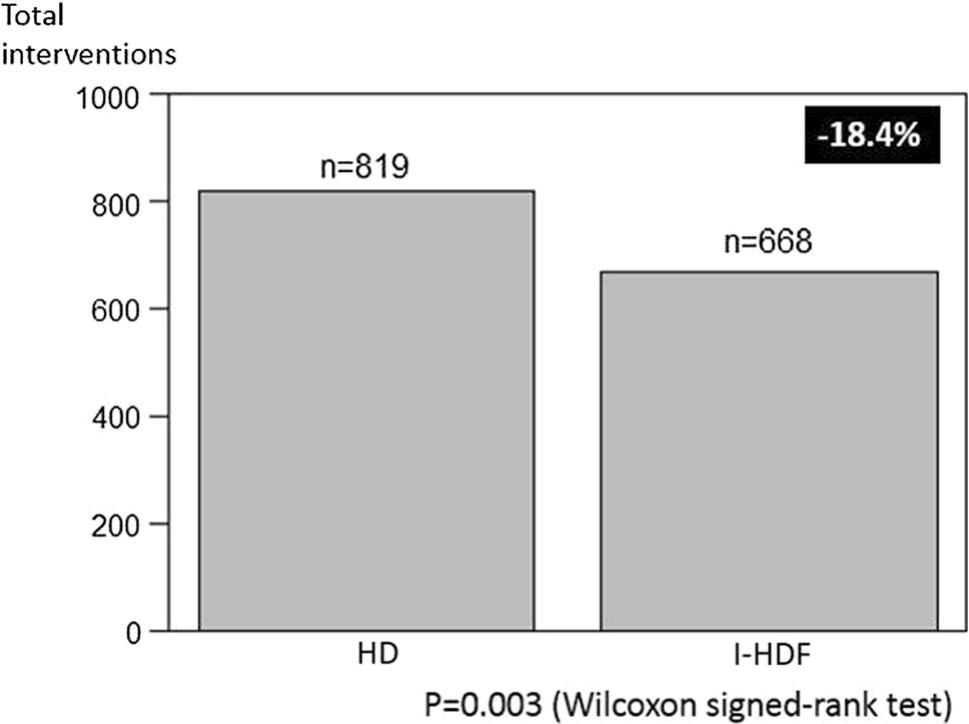
Total number of IDH-related medical and nursing interventions in both HD and I-HDF periods (/816 sessions). *P* value was obtained by the comparison of paired data using Wilcoxon signed-rank test

**Fig. 3:**
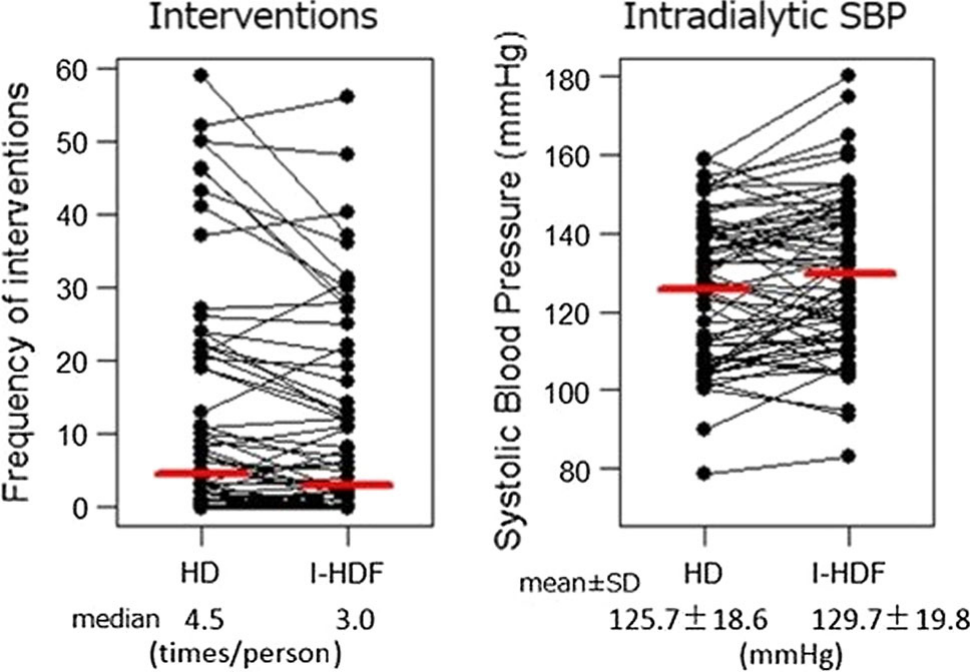
Paired data of frequency of interventions (*times/person/month*, *P* = 0.003, Wilcoxon signed-rank test) and intradialytic systolic blood pressure; SBP (mmHg, *P* = 0.006, paired *t* test)

Table 3 summarizes the data from the I-HDF periods compared to the HD data, including the primary outcomes in patients who completed the study. A total of 819 interventions were registered during 816 sessions of HD. The total interventions reduced from 819 to 668 during I-HDF (−18.4 %, see Fig. 2), from a median frequency of 4.5 to 3.0 times per person (Wilcoxon signed-rank test, *P* = 0.003, see Fig. 3). Details of interventions during the each 4-weeks period (frequency, HD vs. I-HDF) were bolus infusion (27 vs. 24), leg raising with Trendelenburg position (181 vs. 129), lowering ultrafiltration rate (82 vs. 54), switching to sole ultrafiltration (11 vs. 14), discontinuation of session (9 vs. 9), prescription or modification of vasoconstrictor and antihypertensive agents (318 vs. 271) and others (191 vs. 167).

**Table 3:** Interventions, blood pressure and laboratory data in cross-over treatment completed cases

Parameter	HD (*n* = 68)	I-HDF (*n* = 68)	*P* value	Statistics
No. of sessions	816	816		
No. of interventions	819	668	0.003	*^,^^###^
Interventions per person (median [IQR])	4.5 [0.8–20.3]	3.0 [0–13.3]	0.003	*^,^^###^
No. of sessions with hypotension	337	298	0.021	*^,^^###^
Predialysis SBP (mmHg)	134.6 ± 20.8	135.3 ± 23.0	0.774	^#^
Intradialytic SBP (mmHg)	125.7 ± 18.6	129.7 ± 19.8	0.006	*^,^^#^
Postdialysis SBP (mmHg)	130.0 ± 25.2	131.2 ± 26.3	0.592	^#^
DW (kg)	54.9 ± 10.8	54.9 ± 10.8	0.852	^#^
Predialysis weight (kg)	57.0 ± 11.1	57.1 ± 11.2	0.534	^#^
Postdialysis weight (kg)	55.0 ± 10.7	54.9 ± 10.7	0.369	^#^
Predialysis PR (beats/min)	75.1 ± 12.5	76.1 ± 12.3	0.628	^#^
Intradialytic PR (beats/min, at 185 min)	73.1 ± 12.8	70.6 ± 13.9	0.007	*^,^^#^
Postdialysis PR (beats/min)	72.4 ± 13.2	71.8 ± 14.5	0.39	^#^
Net UF (L/session)	2.3 ± 0.7	2.4 ± 0.7	0.588	^#^
Interdialytic weight gain (% of DW)	4.3 ± 1.3	4.4 ± 1.3	0.597	^#^
Total back-filtrate infusion (mL/session)	0	1362 ± 121	*p* < 0.001	*^,^^#^
UFR in HD, cUFR in I-HDF (mL/h/kg)	10.3 ± 3.1	19.5 ± 4.2	*p* < 0.001	*^,^^#^
Membrane area (m^2^)	1.68 ± 0.28	1.62 ± 0.27	0.001	*^,^^#^
Membrane material (PS, %)	82.4	100	*p* < 0.001	*^,^^#^
Hematocrit (%)	32.9 ± 3.2	32.4 ± 3.6	0.088	
BUN (mg/dL)	60.6 ± 13.2	61.0 ± 12.5	0.845	
Urea reduction ratio (%)	70.7 ± 7.9	68.3 ± 6.2	*p* < 0.001	*^,^^#^

Values are given in mean ± SD in normally distributed, median [*IQR* interquartile range] in non-normally distributed and % in categorical variables

*DW* dry weight, *PS* polysulfone, *SBP* systolic blood pressure, *PR* pulse rate, *UFR* ultrafiltration rate, *BUN* blood urea nitrogen, *cUFR* corrected UFR in I-HDF (see text)

^###^ Wilcoxon signed-rank test, ^##^ Chi-square test, ^#^ paired *t*-test, * statistically significant

**Fig. 4:**
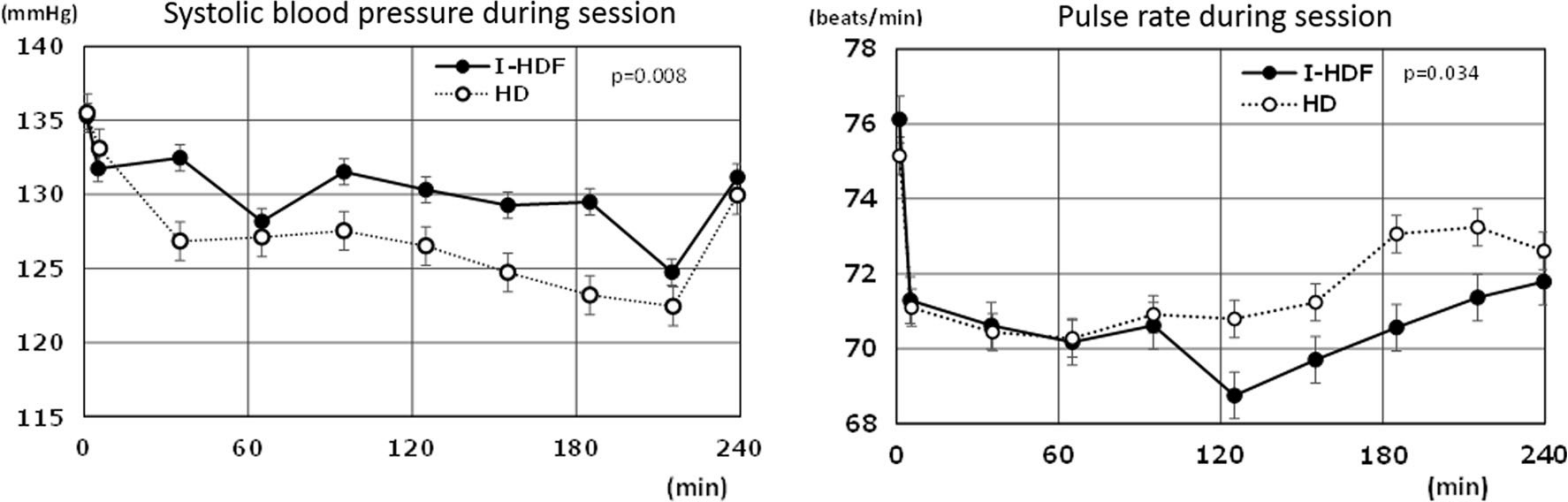
Systolic blood pressure and pulse rate change in HD and I-HDF session; *P* value for the comparison of HD and I-HDF using ANOVA

Along with this, hypotensive sessions also reduced from 337 in HD to 298 in I-HDF (*P* = 0.021). Intradialytic SBP obtained 5 min after infusion increased by 4 mmHg in average (*P* = 0.006, see Fig. 3), in spite of the absence of any change in pre- and post-SBP. Dry weight, pre- and post-body weight did not change throughout the study period. Very importantly, intradialytic pulse rate was lower in I-HDF (see Fig. 4, *P* = 0.007). Membrane area and material were significantly different, but this was due to protocol compliance.

The data were reanalyzed finally with inclusion of data from the six patients who dropped out during the I-HDF period after assigning these subjects the median intervention score of the worsened subjects. In this final analysis the reduction in intervention frequency remained significant (*P* = 0.041).

### Predictive factor for I-HDF responder

To elucidate the characteristics of improved cases for I-HDF, the patients with reduced intervention frequency were treated as responders (*n* = 34), and the patients with the same or increased frequency as non-responders (*n* = 34) (see Table 4).

**Table 4:** Characteristics of responders and non-responders to I-HDF

	Responders (*n* = 34)	Non-responders (*n* = 34)	*P* value	Statistics
Delta frequency of intervention [IQR]^a^	−4 [−8, −2]	1 [0, 1]		
Age (years)	68.6 ± 10.7	63.6 ± 11.3	0.066	^#^
Male (%)	50.0	52.9	0.808	^#^
Diabetics (%)	38.2	26.4	0.299	^##^
Vintage (years; median [IQR])	4.0[2.0–8.8]	4.7[1.9–12.8]	0.605	^###^
Dry weight (kg)	53.8 ± 9.3	55.6 ± 12.3	0.479	^#^
Serum albumin (g/dL)	3.55 ± 0.41	3.62 ± 0.36	0.453	^#^
Cardiovascular disease (%)	47.1	35.3	0.324	^##^
Antihypertensive agent (%)	47.1	52.9	0.628	^##^
Pressor agent (%)	38.2	14.7	0.028	*^,^^##^
Predialysis SBP (mmHg)	136.2 ± 22.8	134.4 ± 23.7	0.751	^#^
Intradialytic SBP increment (mmHg)	6.9 ± 12.4	0.9 ± 9.8	0.029	*^,^^#^
Pulse rate at 35 min (beats/min)	70.3 ± 14.4	71.0 ± 11.0	0.821	^#^
Net UF (L/session)	2.46 ± 0.78	2.17 ± 0.68	0.106	^#^
cUFR (mL/h/kg)	20.6 ± 3.4	18.3 ± 4.6	0.032	*^,^^#^
Interdialytic weight gain (% of DW)	4.8 ± 1.1	4.0 ± 1.5	0.017	*^,^^#^
Membrane area (m^2^)	1.69 ± 0.28	1.67 ± 0.29	0.730	^#^
Hematocrit (%)	32.6 ± 4.1	32.2 ± 3.0	0.599	^#^
BUN (mg/dL)	59.6 ± 12.2	62.3 ± ±12.8	0.382	^#^
Urea reduction ratio (%)	71.9 ± 5.0	69.5 ± 7.1	0.123	^#^
Blood flow (mL/min)	200 [200–200]	200 [200–200]	0.987	^###^
Session time (min)	239.1 ± 11.6	229.4 ± 22.0	0.017	*^,^^#^
Dialysate buffer (citrate/acetate)	17/17	15/19	0.808	^##^

Values are given in mean ± SD in normally distributed, median [*IQR* interquartile range] in non-normally distributed and % for categorical variables

*BV* blood volume, *cUFR* corrected UFR in I-HDF (see text)

^###^ Wilcoxon signed-test, ^##^ Chi-square test, ^#^ *t*-test, * statistically significant

^a^(Interventions during I-HDF) − (interventions during HD)

In univariate comparative analysis, intradialytic SBP rise during HD was 6 mmHg greater in responders. The decrease in interventions thus was paralleled by the observation of greater blood pressure elevation. The treatment time, IDWG and the pressor agent prescription were significantly different between the groups. The corrected UFR was unexpectedly greater in responders. Although not significant, age was suspected to have any effect (*P* = 0.066).

Based on the above result, multiple logistic regression analysis with 7 variables was performed. In addition to the variables having a low *P* value in the univariate comparative analysis, the variables we wish to know its clinical association were selected. The analysis revealed that patients who were older and those with greater IDWG had significant benefit (see Table 5).

**Table 5:** Predictors associated with intervention-reduction by multiple logistic regression analysis

Parameter	Estimate	*P*-value	OR	95 % CI of OR
Age (years)	0.080	0.016	1.083	1.019–1.161*
Interdialytic weight gain (%)	0.641	0.018	1.899	1.162–3.401*
Pressor agent	1.262	0.090	3.531	0.885–17.31
Session time (h)	1.970	0.143	7.169	0.640–189.2
Hemodialysis vintage (years)	−0.075	0.149	0.928	0.827–1.019
History of CVD	−0.564	0.407	0.569	0.138–2.065
Diabetics	−0.338	0.634	0.713	0.171–2.873

We can say that 1 year increase in age, the odds of intervention-reduction by I-HDF (vs. not reduced) increase by a factor of 1.083 and 1 % increase in IDWG (% of dry weight), the odds increase by a factor of 1.899

Likelihood ratio test, *P* = 0.005

*CVD* cardiovascular disease, *OR* odds ratio, *CI* confidence interval

* Statistically significant

## Discussion

IDH is very common, occurring in 20–30 % in chronic hemodialysis patients [1, 9]. Recently, attention has centered on whether IDH can cause organ ischemic damage, such as to the heart muscle and brain [11]. If IDH is repetitive, it impairs a patient’s quality of life, adequate dialysis dose, and organ/peripheral circulation, finally affecting mortality rate [4]. Although risk factors have not been clearly defined, older age, diabetes, cardiovascular illness, autonomic dysfunction, poor nutritional status, severe anemia, dialysate composition, and large IDWG (massive ultrafiltration volume) are considered predispositions for IDH [11]. Altered ET-1 levels [12] and heart rate variance [13] are also involved in the pathogenesis of hypotension during HD.

Among these risk factors, ultrafiltration leading to hypovolemia is the most essential and important factor causing IDH during hemodialysis [1, 2, 6]. Recently, much attention has been given to the convective method, which has a beneficial effect on blood pressure. Locatelli et al. reported that convective therapy reduced intradialytic hypotension, especially the use of on-line predilution HDF [14]. They speculated that the convective mode may have a positive sodium balance and also reduce core temperature which would, contribute to the results, but the true mechanism was still inconclusive.

We centered on a volume-modifying approach, namely intermittent back-filtration infusion HDF, or I-HDF. Mineshima and Eguchi first proposed the method in 2013 [7]. In addition to its anti-hypotensive effect, it improves peripheral circulation monitored by laser flow meter, and increases solute removal, probably due to volume-based vasodilation [7]. Intentional back filtration is performed by a programmed automated dialysis machine with a relatively long cycle (every 30 min), thus the membrane is backwashed to recover some fouling. Low molecular weight protein clearance is maintained better by I-HDF than by conventional continuous filtration. Thus, I-HDF has two points of action: peripheral circulation improvement and membrane fouling prevention [7].

The main finding of this study is the demonstration of lower intervention frequency for IDH in hypotension-prone patients during the I-HDF period (see Figs. 2, 3). Interventions were reduced −18.4 % in total, 4.5–3.0 (per person-month) in median. The lower frequency was clearly associated with a significant increase of intradialytic SBP (see Tables 3, 4). Intermittent infusion irrespective of blood pressure might have mitigated asymptomatic hypotension occurring more frequently than we expected [15].

In addition, pulse rate was significantly lower in I-HDF, suggesting a less sympathetic stimulation (see Fig. 4). Here, we must emphasize that the pulse rate data indicate that I-HDF maintained sympathetic stability. Ultrafiltration increases activity of the sympathetic system and induces compensatory vasoconstriction. Vasoconstriction decreases the dermal tissue circulation and, as a result, core body temperature tends to increase, leading to a higher likelihood of acute hypotension [16]. In physiological conditions, dermal blood flow fluctuates very dynamically. Excessive arteriolar vasoconstriction mediated by sympathetic activation may stimulate the production of adenosine, an endogenous vasodilator [17], and its implication is that hypotension may develop suddenly even when blood volume is still maintained. In addition, a decrease in sympathetic stimulation should increase arteriolar blood flow exponentially [18] and thus improve solute exchange in the capillary bed which should improve final solute removal in I-HDF. Thus, weak sympathetic stimulation in I-HDF should improve cardiovascular stability, prevent ischemic organ injury, and enhance solute removal through capillary and dialysis membranes.

Anti-hypotensive effect of I-HDF was more pronounced in older patients and in patients exceeding a certain level of volume-gain, when analyzed by multiple logistic regression. This may be explained partly by the hypothesis that selected younger, hypotension-prone, patients with less weight-gain may have more complicated problems than mere volume issues, and these problems were not corrected by infusion. The possibility of confounding by unmeasured or unknown factors cannot be excluded. For example, although eating during dialysis may be an important trigger for IDH, we did not survey the relation between them in the present study.

Higher UFR was associated with greater risk of cardiovascular mortality in the hemodialysis (HEMO) study patients [6]. The corrected UFR during I-HDF increased to a range associated with increased risk in that report. However, an important difference to note is that I-HDF is characterized by intermittent infusion, namely intermittent extracellular fluid expansion and blood pressure increment.

A weakness of this study is its relatively small sample size. Although the results obtained are statistically significant, it is necessary to establish that I-HDF really improves comorbidity and survival in a greater number of patients. Further technical improvement is required, such as eventual incorporation of a biofeedback system [5, 19], and replacement volume should be standardized by body size.

In studies involving the treatment of sick people, a substantial dropout rate can be anticipated. There were nine dropouts (11 %) in this study, but we eventually included six who stopped during I-HDF as worsened cases, irrespective of the reason the individual dropped out. However, we must be cautious in interpreting the clinical results when dropouts are present in a crossover study.

A strength of the study is that the observed reduction in intervention correlates with the observation of increased blood pressure during treatment. It gives validity to the results and supports the usefulness of I-HDF as a countermeasure to IDH. The hemodiafilter membrane material was also matched during I-HDF (polysulfone). This should exclude the possibility that our results were influenced by membrane differences that occurred during I-HDF.

Extrapolating from the results of this multicenter study, we may hypothesize that modifying the infusion rate and infusion frequency during treatment of hypotension-prone patients, especially in the case of seniors (around 69 years) with modest ultrafiltration volume (4.8 % of post dialysis weight) may yield further benefits. Finally, I-HDF is economical because it requires no medications.

## Conclusion

I-HDF could be a measure to reduce the need for medical intervention and nursing care in some patients with IDH, and associated with raising intradialytic systolic blood pressure while decreasing sympathetic stimulation. The results imply that the current practice of ultrafiltration during dialysis still needs refinement.
